# Gender- and sex-dependent variations in heart failure and cardiomyopathies: a review of the literature

**DOI:** 10.1007/s00210-026-05029-x

**Published:** 2026-02-14

**Authors:** Tobias Lerchner, Svenja Roß, Florian Buehning, Julia Vogel, Tienush Rassaf, Lars Michel

**Affiliations:** 1 Department of Cardiology and Vascular Medicine, West German Heart and Vascular Center, University Hospital Essen, Hufelandstr. 55, 45147 Essen, Germany; 2 West German Amyloidosis Center Essen, University Hospital Essen, Essen, Germany

**Keywords:** Cardiomyopathies, Gender, Heart failure, Personalized medicine, Sex

## Abstract

Heart failure (HF) is a clinical syndrome that can present as an acute or chronic condition resulting from various cardiovascular (CV) diseases. It poses a significant global health burden and affects individuals regardless of biological sex. HF can be caused by various differing etiologies exhibiting different rates of disease progression and mortality. Treatment of HF is often approached using a one-size-fits-all strategy that overlooks sex and gender differences, relying solely on left ventricular ejection fraction as the clinical parameter. This is due to a lack of evidence and subsequent missing deepened understanding of nuances of disease manifestation, course, and outcome. Biological sex (defined as biological aspects of having female or male body) and gender (society norms and roles of women and men) are known to substantially influence prognosis, age of disease onset, as well as severity in CV disease and subsequent HF. However, varying quality and availability of supporting evidence and subsequently unmet diagnostic and therapeutic needs exist for general HF and cardiomyopathy (CM) management. This review aims to elucidate the influence of sex and gender on the development and treatment of HF and CM as a basis for advancing personalized diagnosis and treatment strategies.

## Introduction

Heart failure (HF) is a clinical syndrome caused by structural and/or functional abnormalities of the heart and presents with various symptoms (e.g., fatigue, dyspnea) and signs (e.g., peripheral edema) (McDonagh et al. 2021; McDonagh et al. 2023). HF presents with various clinical phenotypes. Common causes include ischemic heart disease and cardiomyopathies (CMs) (McDonagh et al. 2021; McDonagh et al. 2023). Adequate diagnosis of underlying pathology is substantial for adequate treatment in a highly variable cohort of patients with HF.

HF is divided into acute (AHF) or chronic (CHF). It can present as HF with preserved (HFpEF), mildly reduced (HFmrEF), or reduced (HFrEF) ejection fraction (McDonagh et al. 2021, 2023). For definite diagnosis of HF, signs including laboratory-proven ventricular wall distention using N-terminal natriuretic peptide (NT-proBNP) as a diagnostic marker are required. Left ventricular ejection fraction (LVEF) is routinely assessed using transthoracic echocardiography and is thus subject to substantial variability.

The incidence of HF is strongly associated with age, which serves as the strongest predictor of hospitalization and cardiovascular (CV) death (McDonagh et al. 2021; Pocock et al. 2006). It is estimated that > 10% of people aged 70 or older are affected by HF. However, the age-specific incidence of HF is decreasing in recent years in Europe and North America (Khan et al. 2024), but crude HF incidence continues to rise due to demographic change, subsequently increasing HF-related mortality (McDonagh et al. 2021, 2023; Bozkurt et al. 2025). Concurrently, HF mortality rates have increased since 2012, with higher age-adjusted mortality observed in 2021 compared to 1999 (Bozkurt et al. 2025).

With the advent of early clinical trials and subsequent guideline-based therapies, survival among patients with HF significantly improved until 2012. HF-related mortality was shown to be reversed during 2012 to 2021, with a higher rate of HF mortality in 2021 than in 1999 according to a US general population database study (Sayed et al. 2024). In dependence on underlying etiology, therapeutic options are limited and often focus on general HF therapy without respecting the underlying heterogeneity in special diseases. In the era of personalized medicine, it becomes increasingly evident that sex plays a pivotal role in reducing overall HF-associated disease load and healthcare strain (Dewan et al. 2019; Aimo et al. 2024a; Olivotto et al. 2005). Evidence accumulated showing differences in onset, prevalence, and outcomes between males and females with HF due to CMs or ischemic heart disease (McDonagh et al. 2021, 2023; Byrne et al. 2023).

## Sex and gender differences in chronic heart failure

HF is ubiquitous distributed between males and females, as shown by landmark studies (Solomon et al. 2024; Anker et al. 2021). However, the etiology of HF, onset, and progress of associated symptoms substantially vary between both sexes.

Despite the substantial impact of several widely cited studies, the proportion of men and women included varied, with a frequently noted underrepresentation of women (Table 1) (Zannad et al. 2011; Maurer et al. 2018; Fontana et al. 2025). Consequently, the evidence supporting current optimal medical therapy in HF is largely based on male-dominant study populations, as noted in current guidelines (McDonagh et al. 2022; Arbelo et al. 2023). Several studies have shown that women receive these recommended therapies at lower rates, despite the absence of sex-specific distinctions in guideline recommendations (Greene et al. 2018; Chandra et al. 2019). In contrast, Ferrari et al. reported that women and men could benefit differently from the same dose of angiotensin receptor neprilysin inhibitors (ARNI). Men showed a reduced risk for HF-caused hospitalization and CV mortality in parallel to an increased dose (Ferrari et al. 2024).

**Table 1:** Summary of landmark trials regarding guideline-directed medical therapy for HF and recent trials demonstrating patient benefit. Relative proportions of included female participants per trial are shown

Trial	*Intervention*	*Entity*	*Female patients (%)*
**ARNI**			
PARADIGM-HF (McMurray et al. 2014)	ARNI vs. Enalapril	LVEF ≤ 40%	21 vs. 22.6
PARAGON (Solomon et al. 2019)	ARNI vs. Valsartan	LVEF ≥ 45%	51.6 vs. 51.8
**ACE-I and AT-1-I**			
SOLVD-Treatment (Investigators et al. 1991)	Enalapril vs. Placebo	LVEF ≤ 35%	19.1 vs. 20.2
SOLVD-Prevention (Investigators et al. 1992)	Enalapril vs. Placebo	LVEF ≤ 35%	11.5 vs. 11.4
SAVE (Pfeffer, et al. 1992)	Captopril vs. Placebo	LVEF ≤ 40%	17 vs. 18
AIRE (Hall et al. 1991)	Ramipril vs. Placebo	LVEF not estimated	27.5 vs. 25.8
ELITE II (Pitt et al. 2000)	Losartan vs. Captopril	LVEF ≤ 40%	30 vs. 31
Val-HeFT (Cohn et al. 2001)	Valsartan vs. Placebo	LVEF ≤ 40%	20.1 vs. 20.0
CHARM-Alternative (Granger et al. 2003)	Candesartan vs. Placebo	LVEF ≤ 40%	31.8 vs. 31.9
CHARM-Added (McMurray et al. 2003)	Candesartan + ACE-I vs. Placebo + ACE-I	LVEF ≤ 40%	21.2 vs. 21.4
CHARM-Preserved (Pocock et al. 2006)	Candesartan vs. Placebo	LVEF ≥ 40%	39.2 vs. 41.0
**Beta-blocker**			
CIBIS-II (The Cardiac Insufficiency Bisoprolol Study II (CIBIS-II) 1999)	Bisoprolol vs. Placebo	LVEF ≤ 35%	19 vs. 20
MERIT-HF (Hjalmarson et al. 2000)	Metoprolol vs. Placebo	LVEF ≤ 40%	23 vs. 22
COPERNICUS (Packer et al. 2001)	Carvedilol vs. Placebo	LVEF ≤ 25%	21 vs. 20
**MRA**			
RALES (Pitt, et al. 1999)	Spironolactone vs. Placebo	LVEF ≤ 35%	27 vs. 27
EMPHASIS-HF (Zannad et al. 2011)	Eplerenone vs. Placebo	LVEF ≤ 35%	22.7 vs. 21.9
**SGLT2-I**			
DAPA-HF (McMurray et al. 2019)	Dapagliflozin vs. Placebo	LVEF ≤ 40%	23.8 vs. 23.0
DELIVER (Solomon et al. 2022)	Dapagliflozin vs. Placebo	LVEF ≥ 40%	43.6 vs. 44.2
EMPEROR-Reduced (Packer et al. 2020)	Empagliflozin vs. Placebo	LVEF ≤ 40%	23.5 vs. 24.4
EMPEROR-Preserved (Anker et al. 2021)	Empagliflozin vs. Placebo	LVEF ≥ 40%	44.6 vs. 44.7
**Vericiguat**			
VICTORIA (Armstrong et al. 2020)	Vericiguat vs. Placebo	LVEF ≤ 45%	24.0 vs. 23.9
VICTOR (Butler et al. 2025)	Vericiguat vs. Placebo	LVEF ≤ 40%	23.8 vs. 23.4
**Digitoxin**			
DIGIT-HF (Bavendiek et al. 2025)	Digitoxin vs. Placebo	LVEF ≤ 40%	19.9 vs. 20.9

*ACE-I* angiotensin-converting enzyme inhibitor, *ARNI* angiotensin-receptor-neprilysin-inhibitor, *AT-1-I* angiotension-1-receptor-inhibitor, *MRA* mineralocorticoid-receptor-antagonist, *SGLT-2-I* sodium-glucose cotransporter 2 inhibitor

Focusing on adverse events following guideline-directed HF medication composed of mineralocorticoid-receptor antagonists (MRA), sodium-glucose cotransporter 2 inhibitor (SGLT2-I), angiotensin-converting enzyme inhibitor (ACE)/angiotensin-1-receptor-inhibitor (AT-1-I), and beta-blocker, women show a pronounced profile of adverse events compared to men in terms of ACE/AT-1-I usage. These findings could not be demonstrated for the use of ARNI (Rambarat et al. 2025). Interestingly, male patients may experience more adverse reactions including hyperkalemia and deterioration of renal function from MRAs (McDonagh et al. 2021; Bots et al. 2019; Kostis et al. 1996; Lopes et al. 2008).

Scoring systems in HF can be substantially impacted by sex and gender, exemplarily, the health-related quality of life (HRQL) and the Kansas City Cardiomyopathy Questionnaire (KCCQ) (Juenger et al. 2002; Jaarsma et al. 2010). Women tend to have a worse HRQL or KCCQ score (i.e., 44% (women) vs. 52% (males)) in patients over the age of 50 compared to men. This difference was observed despite documented signs of HF being similar in women compared to men, and comorbidities were more prevalent in men (Ravera et al. 2021). A higher documentation of severe anxiety or depression in women with HF gives a possible explanation for the greater perceived physiological disability in women (Ravera et al. 2021). Here, geographic localization of included patients did not play a role in the assessed scoring systems (Chandra et al. 2019). The difference of a worse HRQL and KCCQ in women was seen in HFrEF as well as HFpEF patients (Ravera et al. 2021; Teramoto et al. 2022).

Sex differences in clinical presentation also extend to device therapy. Men (44%) are more likely to undergo implantation of implantable cardioverter defibrillator (ICD) or cardiac resynchronization device therapy (CRT) than women (28%) (Chatterjee et al. 2017; Hess et al. 2016).

Studies of CRT and ICD implantation consistently report that women represent a minority of trial participants (25%) (Regitz-Zagrosek 2020; Moss et al. 2002; Kober et al. 2016). However, women generally derive greater benefit from CRT in terms of reduced mortality and HF-related adverse events (Zusterzeel et al. 2014), whereas men tend to experience more favorable outcomes following ICD implantation (Kober et al. 2016). Left ventricular assist devices (LVADs), whether used as a bridge to transplant or as destination therapy for advanced HF, are implanted less frequently in women than in men (Pagani et al. 2009; Varughese et al. 2025). Heart transplantation, the final option for end-stage HF, also reflects this disparity. In 2023, only 27.8% of donor hearts were transplanted into women (Rao et al. 2025). Part of this discrepancy may relate to clinical assessment practices. MacGowan et al. found that women with terminal HF were more likely to be deemed “too well for transplant,” despite meeting criteria for advanced disease (MacGowan et al. 2022). Data on outcomes following heart transplantation as well as potential differences in operative care during heart transplantation is little, but it is known that women experience moderate to severe allograft rejection without difference in mortality compared to men (Hickey et al. 2017).

**Fig. 1:**
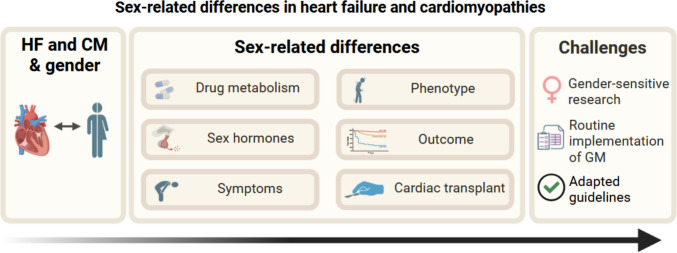
Sex-related differences in HF and CM. Main factors differing between sex and gender in HF and CM and subsequent challenges. Created with BioRender.com. CM, cardiomyopathy; HF, heart failure; GM, gender medicine

Certain pathophysiological mechanisms differ between women and men, particularly regarding pharmacokinetics and the cytochrome P450 (CYP) enzyme family, which is involved in the metabolism of 50% of all drugs (Fig. 1) (Kalibala et al. 2020). CYP activity is frequently higher in women than in men, resulting in increased drug clearance and potentially less sustained therapeutic effects. Evidence further suggests that drug metabolism is hormonally modulated, as estrogen and hormonal contraception can reduce hepatic blood flow and induce specific CYP subclasses, including CYP2A6 and CYP2B6 (Kalibala et al. 2020; Bebawy And Chetty 2009; Spoletini et al. 2012). Regarding other pathophysiological mechanisms associated with HF, including myocardial fibrosis and inflammation, males show more pronounced changes than females (Petrov et al. 2014; Petrov et al. 2010). This difference is hypothesized to result from greater induction of genes encoding components of the renin-angiotensin system in males (Kararigas et al. 2012), eventually leading to enhanced stimulation of cardiac fibroblasts (Dworatzek et al. 2019).

### Acute heart failure and acute coronary syndrome

AHF is a leading cause of hospitalization among individuals over 65 years of age (McDonagh et al. 2021). It is characterized by a sudden and severe onset of symptoms and signs requiring immediate medical intervention (McDonagh et al. 2021). AHF is associated with intra-hospital mortality rates of 4–10% (Crespo-Leiro et al. 2016; Chioncel et al. 2019; Chioncel et al. 2017) and 1-year follow-up mortality of up to 30% (Chioncel et al. 2019; Chioncel et al. 2017). AHF presents either as acute decompensated chronic HF, acute pulmonary edema, isolated right ventricular failure, or cardiogenic shock (CS) (McDonagh et al. 2021, 2023).

CS is a clinical syndrome resulting from severe cardiac dysfunction that leads to inadequate cardiac output, tissue hypoperfusion, and, if untreated, multi-organ failure and death (McDonagh et al. 2021, 2023). The most frequent underlying cause is acute coronary syndrome (ACS), which may present as ST-elevation myocardial infarction (STEMI), non-ST-elevation myocardial infarction (NSTEMI), or unstable angina (Byrne et al. 2023). Evidence indicates that women tend to develop ACS at an older age than men and often present with atypical symptoms, such as mild dyspnea rather than the classic progressive chest pain on exertion (Chandrasekhar et al. 2017). Women are generally more likely than men to seek medical evaluation soon after new symptoms arise but are reported to receive evidence-based treatments less frequently than men (Thompson et al. 2016). This includes timely revascularization during ACS and guideline-recommended medications including angiotensin-converting enzyme inhibitors for HF, statins for secondary prevention, and P2Y12 antagonists after ACS with the need for stent implantation (Bugiardini et al. 2011; Redfors, et al. 2015; Anand et al. 2005; Samayoa et al. 2014).

Myocardial infarction with non-obstructive coronary arteries (MINOCA) represents a clinical entity with distinct pathophysiological and demographic features affecting more women than men in a 1.5:1 ratio as a recent meta-analysis of 15 studies with over 40,000 patients finds (Herrera Flores 2025). MINOCA is associated with the development of LV dysfunction and HFpEF when compared to non-ACS controls (5.3% vs. 1.1%, *p* < 0.001) (Eggers et al. 2019; Almeida 2023), but HF incidence is less than in ACS with obstructive coronary arteries (5.3% vs. 6.3%, *p* = 0.001) (Eggers et al. 2019). One major cause of MINOCA is spontaneous coronary artery dissection (SCAD), a rare but potentially fatal etiology of ACS that predominantly affects young women without traditional CV risk factors or prior HF (Lebrun And Bond 2018). Although the exact incidence of SCAD remains uncertain, it is estimated to account for approximately 1.7–4% of all ACS cases (Rashid et al. 2016; Nishiguchi et al. 2016). SCAD shows a strong association with pregnancy, with up to 40% of cases occurring in the postpartum period (Claudon et al. 1972). While SCAD primarily affects women of childbearing age, its occurrence in men increases in the presence of underlying arteriopathies, such as fibromuscular dysplasia (Lebrun And Bond 2018). The marked female predominance may contribute to underdiagnosis in men, and data on male SCAD cases, and thus reliable evidence, remain limited.

### Sex-specific causes of heart failure

HF in both males and females can arise from sex-specific conditions such as genetic abnormalities or pregnancy-related factors.

Peripartum cardiomyopathy (PPCM) is defined as a form of idiopathic CM characterized by left ventricular (LV) systolic dysfunction leading to HF, occurring toward the end of pregnancy or within several months postpartum. PPCM represents a complex and potentially life-threatening condition, constituting a sex-specific subtype of idiopathic CM in women (Hoes et al. 2022). PPCM is caused by multiple mechanisms, such as genetic mutations in sarcomeric and DNA repair genes, and acquired factors, including increased beta-adrenergic activation. This interplay leads to the generation of oxidative stress with a subsequent change of prolactin into a 16 kDa metabolite, affecting the vasculature and leading to endothelial cell death. Following this, reduced cardiomyocyte metabolism fosters PPCM (Sliwa et al. 2025).

Complementary data from animal models and human induced pluripotent stem cell (hiPSC)-derived cardiomyocytes suggest that disturbances in cellular metabolism and angiogenic signaling are central pathogenic pathways in PPCM, both in vivo and in vitro (Hoes et al. 2020; Patten et al. 2012*).* The hormonal milieu of late pregnancy, particularly maladaptive responses to prolactin cleavage products and associated vascular dysfunction, is considered a key contributor (Hoes et al. 2022).

In men, mosaic loss of the Y chromosome (mLOY) has emerged as a risk factor for CV disease. mLOY increases with age and smoking exposure and is associated with clonal hematopoiesis of indeterminate potential (CHIP) (Dumanski, et al. 2015; Thompson et al. 2019; Ljungstrom et al. 2022). This condition promotes age-related pro-fibrotic remodeling, reduced cardiac function, and elevated mortality (Loftfield et al. 2018). mLOY is further linked to major secondary CV events, including myocardial infarction and stroke (Haitjema et al. 2017). Preclinical studies indicate that mLOY in leukocytes can directly contribute to HF development in men (Sano et al. 2022), suggesting that affected individuals may benefit from targeted antifibrotic therapies (Sano et al. 2022).

In contrast to HF driven by inherently sex-dependent mechanisms such as pregnancy or mLOY, certain conditions can occur in both sexes but show clear sex-related predominance. Takotsubo syndrome, an acute stress-induced CM characterized by transient LV dysfunction, primarily affects postmenopausal women (80–90%) (Omerovic and Redfors 2025; Ghadri et al. 2018; Lyon et al. 2016). Nevertheless, it can develop at any age in response to extreme emotional or physical stress (Omerovic and Redfors 2025; Lyon et al. 2016). Takotsubo syndrome remains frequently underdiagnosed, particularly in patients with concurrent or pre-existing coronary artery disease (CAD) (Ghadri et al. 2018; Lyon et al. 2016). Its pathophysiology involves microvascular dysfunction, catecholamine surges, and psychosomatic factors (Omerovic and Redfors 2025; Deshmukh et al. 2012; Suzuki et al. 2014). Currently, evidence-based management recommendations are limited, as randomized controlled trials (RCTs) are still ongoing (NCT02867878; NCT046664549) (Lyon et al. 2016).

### Heart failure in special populations

With the advent of gender-affirming hormonal therapy, CV risk assessment has gained increasing importance. Hormonal therapy for transgender women typically involves estradiol combined with anti-androgens to suppress testosterone, and testosterone is administered to transgender men (Zijverden et al. 2024; Coleman et al. 2022). The influence of sex hormones on CV outcomes remains a matter of debate; however, evidence suggests that hormonal therapy in both transgender men and women is associated with an approximately 40% increase in the risk of adverse CV events, including myocardial infarction with subsequent development of HF, stroke, and venous thromboembolism (Zijverden et al. 2024; Albrektsen et al. 2016; Arnold et al. 2017). Anti-androgens, commonly used in prostate cancer patients, are known to promote CV events and arrhythmia, but their role in HF is less characterized (Ong et al. 2024; Yaxley And Fitzgerald 2025). Preclinical data indicate a potential role of antiandrogens via inhibition of cardiomyocyte apoptosis and potentiation of fibrosis and, thus, reduced contractility (Sanchez-Mas et al. 2010). Despite this, the rate of HF from anti-androgens is low in recent studies (Khorram et al. 2024; Baser et al. 2024).

HF therapies to women of childbearing potential are challenging due to the high risk of embryotoxicity and fetal safety concerns. Renin–angiotensin–aldosterone-system (RAAS) inhibitors including ACE-I, ARBs, and ARNI are associated with fetopathy following exposure during the second and third trimesters and are therefore contraindicated in pregnancy (Bateman et al. 2017). Although first-trimester risks appear less pronounced, these drugs are commonly avoided or substituted during pregnancy (Bateman et al. 2017). Beta-blockers are generally considered safe for use during pregnancy, but certain agents, particularly atenolol, have been linked to fetal growth restriction and therefore require careful, agent-specific risk–benefit evaluation (Martinez et al. 2023; Duan et al. 2018). Recommendations for MRAs are less clear. Spironolactone is typically avoided due to its anti-androgenic effects on male fetuses, while evidence for eplerenone remains limited, restricting its use to exceptional cases (Riester And Reincke 2015; Balachandran et al. 2024). Data on newer metabolic agents such as SGLT2 inhibitors and glucagon-like peptide-1 receptor agonists are sparse.

Diagnostic imaging considerations in suspected HF during pregnancy are due to dose- and gestational age-dependent radiation-associated risk. Deterministic effects, including congenital malformations and severe neurodevelopmental impairment, generally occur only at doses above 100 milligray (mGy). Diagnostic radiation exposure is therefore minimized based on careful assessment of alternative diagnostic modalities and suspected therapeutic consequences (Saltybaeva et al. 2020). To date, no study has explicitly evaluated whether concerns about fetal radiation exposure lead to underdiagnosis of pulmonary embolism or SCAD as a result of reduced use of diagnostic imaging. Limited evidence indicates that fetal exposure to less than 50 mGy (0.03–0.66 mGy as reported standard range in computed tomography pulmonary angiography) was reported to not be associated with increasing risk for death, malformation, impaired neurodevelopment, or cancer (Leung et al. 2012).

### Cardiomyopathies

CMs are primary diseases of the myocardium with structural and subsequent functional disorders, unexplained by CAD or abnormal loading conditions (Elliott et al. 2008). They can be divided into familial/genetic and non-familial/non-genetic forms; however, dedicated subtypes exhibit a combination of both (Elliott et al. 2008; Verdonschot and Heymans 2024; Authors, et al. 2014). Differences in sex regarding outcomes and phenotypic expressions are well known in CM and CV medicine (Arbelo et al. 2023). Differences in genetics, hormonal levels, physician-dependent management, healthcare access, representation in trials, and therapy response between men and women are contributing factors to the observed disparities in outcomes and diagnostics between the sexes (Arbelo et al. 2023; Pelliccia et al. 2019; Fumagalli and Olivotto 2020).

### Dilated cardiomyopathy

Dilated CM (DCM) is defined as biventricular or left ventricular dilatation and systolic dysfunction in the absence of CAD or abnormal loading (Arbelo et al. 2023; McKenna et al. 2017). It is estimated that 1 in 250 people show either clinically relevant or subclinical DCM manifestation (Hershberger et al. 2013). DCM, despite being a diagnosis of exclusion, is the most common reason for cardiac transplant worldwide.

Sex is a recognized disease modifier for DCM manifestation, but distinct recommendations on how to adapt diagnosis and therapy have not been established. How sex- and gender-related aspects can be embedded in routine clinical management remains a matter of discussion and future research (Arbelo et al. 2023; Heidenreich et al. 2022). Recent landmark studies on HF and DCM included fewer women than men (Kober et al. 2016; Jin et al. 2020; Whitelaw et al. 2021), and reported no sex or gender-specific mortality differences (Kober et al. 2016). There is an ongoing debate regarding the reliability of sex-neutral used clinical imaging parameters (Owen et al. 2024). So far, inter-individual differences, including underlying pathophysiology in DCM, are not addressed by currently used imaging modalities. Myocardial fibrosis, a hallmark of adverse myocardial remodeling in DCM, appears to be more pronounced in symptom-matched women than in men, accompanied by a relatively greater reduction in systolic function (McDonagh et al. 2022; Owen et al. 2024). The pathophysiology of myocardial fibrosis in DCM is based on excessive inflammatory response mediated by the IL-1 cascade, priorly activated through damage- and pathogen-associated molecular patterns with subsequent NLRP3-inflammasome activation in cardiomyocytes (Gigli et al. 2024). These observations explain the paradox of a worse prognosis in women with DCM despite their generally milder symptoms compared to men with a favorable clinical course and better response to HF therapy, lower rates of SCD, and lower post-transplant mortality (Owen et al. 2024; Gigli et al. 2019; D'Amario et al. 2020; Wahbi et al. 2019; Rijsingen et al. 2012; Vissing et al. 2021; Halliday et al. 2018; Herman et al. 2012).

### Hypertrophic cardiomyopathy

Hypertrophic CM (HCM) is a primary disorder of the myocardium defined as LV hypertrophy not solely attributable to external factors including pressure overload (Authors, et al. 2014; Ommen et al. 2020). Patients with HCM experience an increased risk for HF development and malignant arrhythmias with subsequently sudden cardiac death (Ho et al. 2021). HCM can be divided into obstructive (HOCM) and non-obstructive forms depending on peak LV outflow tract obstruction (≥ 30 mmHg).

Sex-related differences in phenotypes and outcomes were extensively studied in patients with HCM/HOCM. Males predominate in a 3:2 ratio regarding HCM incidence and prevalence (Table 2) (Olivotto et al. 2005; Kubo et al. 2010). Further, diagnosis is established earlier in males with a subsequent shorter duration until symptom onset compared to females (Dimitrow et al. 1997). Since guidelines recommend the establishment of HCM diagnosis based on echocardiographic evaluated maximum wall thickness (MWT) ≥ 15 mm, there is a strong suspicion of under- as well as overdiagnosis depending on underlying sex (Huurman et al. 2020). Recently, it was shown that indexation of MWT for body surface area or weight decreased the prevalence of HCM in especially larger men (Huurman et al. 2020). The influence of sex and gender on prognosis in HCM has been the subject of considerable debate. A recent meta-analysis by Trongtorsak et al. summarized data from eleven retrospective cohort studies involving approximately 10,000 patients with HCM (Trongtorsak et al. 2021). Female sex represented a significant risk factor for all-cause and HCM-related mortality as well as rehospitalization due to worsening HF despite lower disease prevalence in aggregate data analysis (Trongtorsak et al. 2021; Zhao et al. 2023). The HCM Risk-SCD score is recommended by current guidelines for predicting SCD, but it does not account for sex (Zhao et al. 2023). Several parameters included in the HCM Risk-SCD score tend to be lower in females, but lower values do not necessarily indicate a lower disease burden. Relying on the HCM Risk-SCD score in clinical practice may therefore inadvertently bias risk estimation in women, particularly in those with borderline diagnostic findings (Arbelo et al. 2023; Ommen et al. 2020).

**Table 2:** Summary of the current known differences and main challenges between men and women in CM and HF

	Main sex-dependent differences	Main challenge
Heart failure (HF)		
Acute HF	Women more often present with atypical symptoms and have higher rates of HFpEF during acute decompensation, while men more often present with acute decompensation from ischemic HF (Regan et al. 2025)	Ensure timely recognition of sex-specific symptom profiles and access guideline-directed therapies
Chronic HF	Women remain underrepresented in most HF RCTs and may show different pharmacokinetic/pharmacodynamic responses to therapy (Lam et al. 2019)	Develop and implement sex-specific treatment recommendations and dosing strategies
HFpEF	Overrepresentation of women (Lam et al. 2019; Khan et al. 2022)	Increase the proportion of women in HFpEF trials to reflect epidemiology
HFmrEF	Men more often have ischemic cause compared to women (Lam et al. 2019)	Generate evidence on sex-dependent treatment effects
HFrEF	Men remain overrepresented in clinical trials (McDonagh et al. 2022)	Improve data on optimal medical therapy in women
HFimpEF	Women more frequently show EF improvement (Kewcharoen et al. 2022)	Develop sex-specific follow-up and long-term treatment strategies
Ischemic HF	Women show more coronary microvascular dysfunction; men experience earlier onset of disease due to earlier onset of CAD (Lam et al. 2019)	Improve early diagnosis and treatment of ischemia in women
HF due to myocarditis	Men exhibit more fulminant courses. Women more often show autoimmune forms (Fairweather et al. 2023)	Increase data on sex-dependent LGE patterns and immune signatures in CMR/biopsy
HF due to ICI-therapy	Women seem to have a higher susceptibility for ICI-myocarditis (Yousif et al. 2023)	Develop sex-stratified monitoring protocols and risk assessment tools
HF due to anthracyclines	Pre-menopausal women may have lower vulnerability to LV dysfunction (Wilcox et al. 2022)	Identify biomarkers for sex-dependent monitoring strategies
HF due to valvular pathologies	Men develop more fibro-calcific lesions (Grego et al. 2025). Women show higher incidence of paradoxical low-flow aortic stenosis (Appleby et al. 2024)	Validate diagnostic cut-offs specifically for women
Cardiomyopathies (CM)		
DCM	Sex and gender influence phenotypic presentation. Women often exhibit more severe remodeling but have milder clinical presentations	Adjust diagnostic thresholds (i.e., LVEF, LV dimensions) to account for sex-dependent physiology and clarify sex-specific impact on outcomes
HCM	Men are diagnosed more frequently (approx. 3:2), whereas women often present later and with more advanced symptoms despite similar genetic backgrounds (Meyer et al. 2014)	Establish improved sex-specific echocardiographic cut-offs for maximal wall thickness and refine early detection in women
RCM	Apparent male predominance (around 90%) may reflect diagnostic bias rather than true prevalence (Vogel et al. 2025)	Determine the true sex distribution and identify optimal sex-adapted diagnostic and treatment pathways
ARVC	Men show higher penetrance and more frequent arrhythmogenic events, whereas women may remain longer with a subclinical phenotype despite carrying identical variants (Meyer et al. 2014)	Enable earlier identification of subclinical disease in both sexes to prevent sudden cardiac death as first manifestation
Takotsubo CM	Predominantly affects postmenopausal women (approx. 9:1 female-to-male ratio), possibly due to hormonal susceptibility to catecholamine surges (Omerovic and Redfors 2025)	Develop targeted treatment strategies from emerging RCT evidence and improve diagnostic criteria and clinical awareness
Peripartum CM	Associated with genetic variants shared with DCM (i.e., *TTN*), with unique pregnancy-related triggers (Bauersachs et al. 2019)	Clarify underlying pathophysiology and identify targeted therapies to improve maternal outcomes
Toxic CM	Men have higher prevalence of alcohol-toxic CM	Identify sex-specific mechanisms driving susceptibility to toxic CM
Inflammatory CM	Men are more likely to develop progressive ventricular remodeling and systolic dysfunction, whereas women more often retain preserved ventricular function despite inflammation (Fairweather et al. 2023; Heymans et al. 2023)	Improve diagnostic methods accounting for sex-dependent immune responses

*ARVC* arrhythmogenic right ventricular CM, *CAD* coronary artery disease, *CM* cardiomyopathy, *CMR* cardiac magnetic resonance imaging, *DCM* dilated CM, *HCM* hypertrophic CM, *HFimpEF* HF with improved ejection fraction, *HFmrEF* HF with midrange ejection fraction, *HFpEF* HF with preserved ejection fraction, *HrEF* HF with reduced ejection fraction, *ICI* immune checkpoint inhibitor, *LGE* late gadolinium enhancement, *LV* left ventricle, *LVEF* left ventricular ejection fraction, *RCM* restrictive CM, *RCT* randomized controlled trial.

Guideline-directed therapy for HCM/HOCM focuses on alleviating HF symptoms, performing invasive septal reduction when indicated, and, more recently, employing cardiac myosin inhibitors such as mavacamten (Ommen et al. 2020; Maron et al. 2006; Olivotto et al. 2020; Desai et al. 2022; Masri et al. 2024). Myosin inhibitors target core disease mechanisms, leading to reductions in diastolic dysfunction and hypercontractility and consequently decreasing disease-specific HF burden (Masri et al. 2024). Recent clinical trials of mavacamten and the candidate-drug aficamten enrolled balanced numbers of male and female participants (Olivotto et al. 2020; Masri et al. 2024; Ho et al. 2020). Although sex-specific subgroup analyses were limited, mavacamten demonstrated superior efficacy compared with placebo in both sexes. Notably, women exhibited a more pronounced improvement in KCCQ scores, suggesting a greater perceived quality-of-life benefit (Olivotto et al. 2020). It is known that women tend to have increased CYP enzyme activity, which may influence mavacamten metabolism. To date, there is no evidence indicating whether these sex-related differences in CYP activity affect mavacamten pharmacokinetics. In contrast, the metabolism of the candidate drug aficamten is less CYP-dependent, which could represent a potential benefit in drug efficacy.

### Restrictive cardiomyopathy including cardiac amyloidosis

Restrictive CM (RCM) is defined as restrictive left and/or right ventricular function with concomitant normal or reduced systolic or diastolic capacity regardless of ventricular wall thickness (Elliott et al. 2008). RCM is a heterogenous group of primary and secondary (endo-) myocardial phenotypes with different prevalence, depending on sex or gender, ethnicity, and age.

The most common cause of RCM is cardiac amyloidosis (CA), which is caused by the accumulation of misfolded protein fragments in the extracellular space (Siddiqi And Ruberg 2018). Early recognition and subsequent treatment of CA remain a problem in routine clinical practice, as awareness remains low and screening parameters are non-specific (Vogel et al. 2025; Lane et al. 2019; Grogan et al. 2016). The combination of clinical symptoms suggestive of HF, an increase in interventricular wall thickness ≥ 12 mm, and the presence of at least 1 red flag sign/symptom should lead to further screening for CA (Vogel et al. 2025; Gonzalez-Lopez et al. 2017; Garcia-Pavia et al. 2021). Women, in general, tend to have a lower absolute interventricular septum, posterior wall thickness, and LV end-diastolic diameter than men and thus are less likely to get diagnosed. However, when indexed, wall thickness is equal or even increased in women (Aimo et al. 2024b). Based on these findings, a profound sex or gender imbalance in the prevalence of cardiac amyloidosis has been observed, with approximately 88–92% of patients with ATTRwt-CM being male (Antonopoulos et al. 2022). In parallel, current therapeutic studies for the treatment of ATTRwt included between 87 and 91% of male patients (Maurer et al. 2018; Fontana et al. 2025; Vogel et al. 2025; Gillmore et al. 2024), thereby questioning the efficacy and safety of available pharmaceutical therapy approaches for women. More scientific evidence, including risk factors, clinical characteristics, biomarkers, and imaging, is needed to target the diagnostic gap of CA in women (Arbelo et al. 2023). Additionally, information on differences in periprocedural safety of endomyocardial biopsy (differences in cardiac wall thickness), and on long-term differences in available treatments is required. It will be key to enhance general awareness and diagnostic accuracy to be able to treat women affected by CA faster and more precisely.

### Arrhythmogenic right ventricular cardiomyopathy

Arrhythmogenic right ventricular CM (ARVC) is an inherited CM based on an underlying disease-causing genotype, which most commonly affects the plakophilin-2 gene (PKP2) and often manifests with effort-induced syncope and sudden cardiac death (SCD) (Oomen et al. 2018). Mutations in PKP2 lead to a fibrotic or fibrofatty remodeling of right- or bi-ventricular myocardium, with subsequent progressive dysfunction (Prior And La Gerche 2020; Alencar, et al. 2018). ARVC is considered a rare cause of HF, with an estimated prevalence of 1 in 2500–5000 people, but accounts for 5–10% of SCD in athletes under the age of 65 (James And Calkins 2019; Alblaihed et al. 2023; Corrado et al. 2017).

Currently, cardiac transplant remains the only curative treatment option available for ARVC. Drug-based therapy is based on general HF and antiarrhythmic therapy including ICD implantation. To date, gene-based treatment options are intensely discussed with various preclinical trials aiming to change guidelines and best clinical practice in the future. The Pkp2 gene therapy drug RP-A601 is now evaluated in a phase 1 trial (NCT05885412) and is reported to reduce ventricular arrhythmia, reverse right ventricular remodeling, improve cardiac function, and prolong survival in a mouse model of ARVC (Linthout, et al. 2025).

ARVC exhibits a higher prevalence in males than in females, with an approximate male-to-female ratio of 3:1 (Pelliccia et al. 2019). Sex is linked to differences in ARVC manifestation, including variable genetic penetrance and phenotypic expression. Hormonal influences appear to contribute to these disparities, as elevated serum testosterone levels in men and reduced estradiol levels in women have been associated with adverse outcomes (Akdis et al. 2017). Male patients demonstrate higher incidence-adjusted mortality and SCD rates than females and tend to exhibit more severe arrhythmic manifestations. A more advanced phenotype in men is further indicated by larger right ventricular (RV) volumes, reduced RV ejection fraction, and greater LV involvement (Lin et al. 2017). In addition, men more frequently present with ventricular tachycardia (VT) or ventricular fibrillation (VF) as initial manifestations of the disease. Electrocardiographic abnormalities characteristic of ARVC, such as T-wave inversions in the precordial leads, epsilon waves, or RV conduction delay, are also more common in men, which may reflect more extensive myocardial involvement but may also help to guide diagnosis (Meyer et al. 2014).

Although men are more frequently and severely affected, ARVC in women is often underdiagnosed. Female patients more commonly present with subclinical or mild forms of the disease, which further challenges timely and accurate diagnosis (Pelliccia et al. 2019). To date, the cause of these sex differences is not fully understood, but differences in physical exercise between men and women as well as the hormonal milieu in terms of sex-hormones might play a role (Meyer et al. 2014; Akdis et al. 2017).

### Sex and gender in research of heart failure and cardiomyopathies

In HF research, the incorporation of sex as a biological variable and the explicit use or definition of the term gender remain inconsistent (Regitz-Zagrosek 2020; Luscher 2020; Blumer et al. 2024). Literature points out that “sex” and “gender” are used interchangeably even though sex (biological attributes) and gender (sociocultural/behavioral attributes) represent distinct constructs (Blumer et al. 2024; Regitz-Zagrosek And Gebhard 2023). Further, explicit definitions of either term or their handling in the dedicated study design are often missing (Regitz-Zagrosek And Gebhard 2023). Consequently, recommendations have emerged for more rigorous study design, thus ensuring adequate enrolment of both sexes, sex-based analyses, and clearly defined status of sex and gender (Luscher 2020; Blumer et al. 2024).

Sex-related restrictions in preclinical research models remain a major issue regarding the translation of data to women. In animal models of ischemia–reperfusion (I/R) injury, male animals remain predominantly studied (Ostadal and Ostadal 2014; Wever et al. 2015; Rigby et al. 2020). This imbalance is commonly reasoned by suspected sex-specific differences in infarct size, as female animals may exhibit smaller infarcts and superior functional recovery (Ostadal and Ostadal 2014; Medzikovic et al. 2023). Exemplarily, a difference in the range of 25% to up to twofold smaller infarcts in female compared with male mice was reported (Guo et al. 2012; Bae And Zhang 2005; Bouma et al. 2010). Female mice exhibit reduced myofibroblast differentiation and collagen production, resulting in less fibrotic remodeling (Pullen et al. 2020). They also show fewer pro-inflammatory infiltrating monocytes and higher levels of anti-inflammatory monocytes (Pullen et al. 2020; Johnson et al. 2006). In addition, the number of mitochondria is lower in female than in male cardiomyocytes (Colom et al. 2007). Female cardiomyocytes display greater resistance to mitochondrial swelling and a higher degree of post-translational modifications in ROS-regulating and ROS-producing enzymes (Ostadal et al. 2019; Ostadal et al. 2009). Consequently, a substantial sex bias persists in preclinical I/R studies designed to model ischemic HF when restricting to male animals.

## Conclusion and future perspective

Gender and sex are important yet often overlooked factors in HF and CMs, influencing pathophysiology, incidence, disease presentation, risk factors, outcomes, and response to therapy. The unequal sex and gender distribution in clinical studies has frequently led to imbalanced evidence which is insufficiently addressed in everyday clinical practice. Only a few studies acknowledge the profound difference between biological sex and gender. A more nuanced understanding of gender-specific differences, along with the development and implementation of gender-sensitive diagnostic and therapeutic algorithms, could significantly improve patient care in the future.

## Data Availability

All source data for this work (or generated in this study) are available upon reasonable request.
